# Supplementary material for: Nuclear Lipid Droplet Birth during Replicative Stress
Source: Cells. 2022 Apr 20;11(9):1390. doi: 10.3390/cells11091390 (PMC9105094; doi:10.3390/cells11091390)
Supplement: Supplementary file 1 [file cells-11-01390-s001.zip › Supplementary Material Kumanski et al REVISED/Figure S1.pdf]

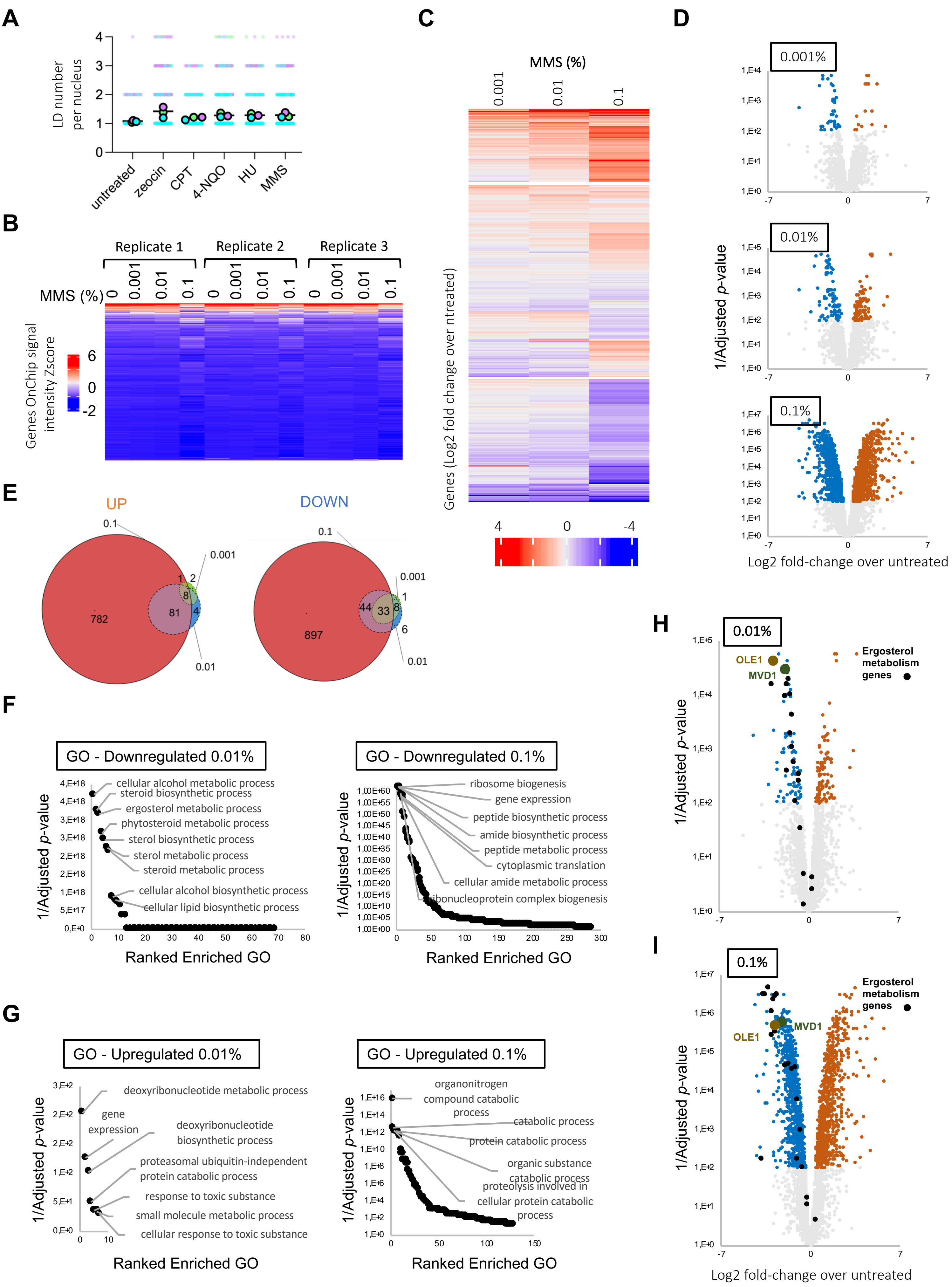
A
LD number per nucleus
untreated
zeocin
CPT
4-NQO
HU
MMS
B
Replicate 1
Replicate 2
Replicate 3
MMS (%)
0
0.001
0.01
0.1
Genes OnChip signal intensity Zscore
6
0
-2
C
MMS (%)
0.001
0.01
0.1
Genes (Log2 fold change over ntreated)
4
0
-4
D
0.001%
0.01%
0.1%
1/Adjusted p-value
Log2 fold-change over untreated
E
UP
DOWN
782
81
8
1
2
4
897
44
33
8
1
6
F
GO - Downregulated 0.01%
cellular alcohol metabolic process
steroid biosynthetic process
ergosterol metabolic process
phytosteroid metabolic process
sterol biosynthetic process
sterol metabolic process
steroid metabolic process
cellular alcohol biosynthetic process
cellular lipid biosynthetic process
Ranked Enriched GO
GO - Downregulated 0.1%
ribosome biogenesis
gene expression
peptide biosynthetic process
amide biosynthetic process
peptide metabolic process
cytoplasmic translation
cellular amide metabolic process
ribonucleoprotein complex biogenesis
G
GO - Upregulated 0.01%
deoxyribonucleotide metabolic process
gene expression
deoxyribonucleotide biosynthetic process
proteasomal ubiquitin-independent protein catabolic process
response to toxic substance
small molecule metabolic process
cellular response to toxic substance
GO - Upregulated 0.1%
organonitrogen compound catabolic process
catabolic process
protein catabolic process
organic substance catabolic process
proteolysis involved in cellular protein catabolic process
H
0.01%
OLE1
MVD1
Ergosterol metabolism genes
I
0.1%

Figure S1. MMS treatment triggers a downregulation of the fatty acid unsaturation and sterol transcriptional network

A. The number of lipid droplets in individual nuclei of *S. cerevisiae* cells treated as indicated was counted and plotted. Each dot of a light color refers to one nucleus. At least 300 nuclei were considered per condition and experiment. Three independent experiments were performed. To account for reproducibility, each experiment is shown in a different color. Darker, bigger dots indicate the mean of each experiment. The horizontal black line indicates the mean of the means.

B. Heatmaps of chip average signal intensity for every gene after normalization and filtering to visualize the three replicates. See also Table S1.

C. Heatmaps of gene expression upon MMS treatment. Scale represents the averaged Log2 fold-change over untreated samples of the three considered independent experiments. See also Table S2-4.

D. Volcano plots of all genes in response of the three doses of MMS. Fold-changes were computed from the average RNA signal of MMS-treated samples *versus* the average RNA signal of untreated samples. Adjusted *p*-values were computed with the Limma software [9] and corrected for multiple tests. Genes with significant genes according to Adjusted *p*-value are depicted in color, with upregulated ones in orange and downregulated ones in blue. The considered MMS dose in indicated above each graph.

E. Venn Diagrams representing the overlap between the three doses of MMS in absolute numbers. Left: upregulated genes. Right: downregulated genes.

F, G. Overrepresentation test on downregulated genes (F) or the upregulated ones (G) upon 0.01%- and 0.1%-MMS treatment. Enrichment is computed with Fisher's test and corrected for multiple testing by false discovery rate. 1/Adjusted *p*-values for all enriched pathways are represented. The top pathways are annotated. See also Table S5-9.

H, I. Volcano plots of all genes in response to 0.01% MMS and 0.1% MMS. Fold-changes are computed from the average RNA signal of MMS-treated samples versus the average RNA signal of untreated ones. Adjusted *p*-values were computed with the Limma software [9] and corrected for multiple tests. Genes with significant changes according to the adjusted *p*-values are depicted in color, where upregulated ones are shown in orange and downregulated ones in blue. Genes annotated as related to ergosterol metabolism are annotated and depicted in black. *OLE1* and *MVD1* are represented in brown and green, respectively. See also Table S2-4.
